# P-1077. Study of Prescribing patterns and Effectiveness of Ceftolozane/Tazobactam (C/T) Real-world Analysis (SPECTRA): Clinical Outcomes and Treatment Patterns from Mexico

**DOI:** 10.1093/ofid/ofae631.1265

**Published:** 2025-01-29

**Authors:** Ines Saldivar, Pedro Martínez Ayala, Adrian Camacho Ortiz, Paulo Castañeda-Méndez, Rayo Morfin-Otero, Yanbing Zhou, Thales Polis, Emre Yucel

**Affiliations:** Hospital Angeles Metropolitano, CIUDAD DE MÉXICO, Distrito Federal, Mexico; Hospital Country 2000, Guadalajara, Jalisco, Mexico; HOSPITAL UNIVERSITARIO DR. JOSE ELEUTERIO GONZÁLEZ, NUEVO LEÓN, Nuevo Leon, Mexico; Hospital Medica Sur, Tlalpan, Distrito Federal, Mexico; Centro Universitario de Ciencias de la Salud, Universidad de Guadalajara, Guadalajara, Jalisco, Mexico; Merck, Rahway, New Jersey; MSD, Sao Paulo, Rio de Janeiro, Brazil; Merck & Co., Inc., North Wales, Pennsylvania

## Abstract

**Background:**

Reported are results of SPECTRA data from Mexico

**Figure:**
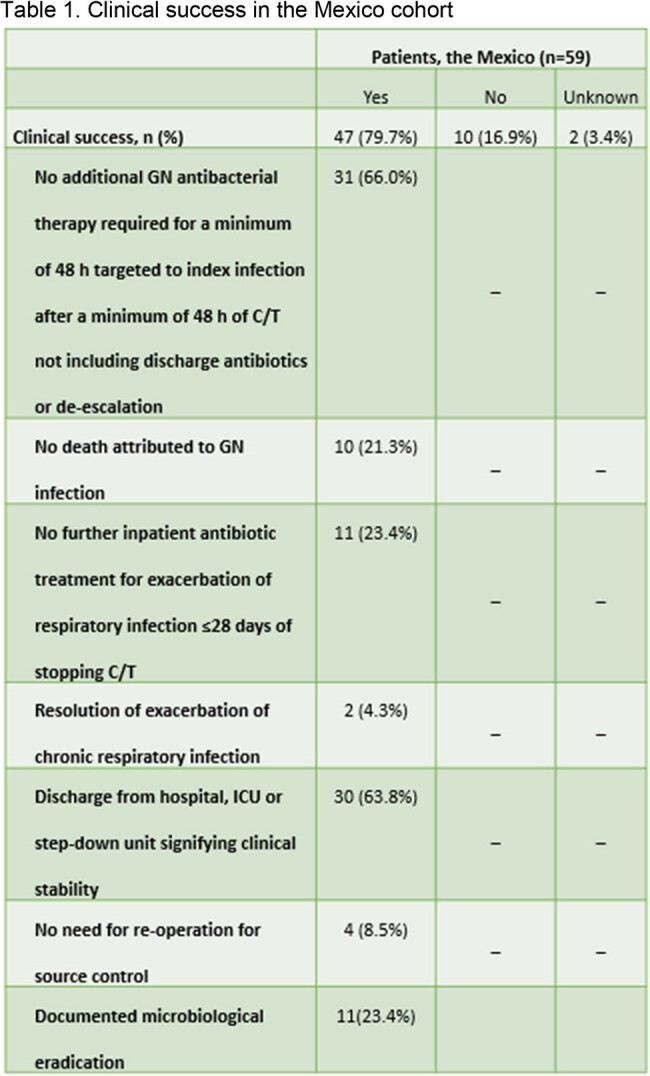

**Methods:**

SPECTRA (n=617) is a 7-country multicentre retrospective observational study, including hospitalized adult patients (≥18 years) treated with C/T for ≥48 hours. Medical records were reviewed for 30 days after the last dose of C/T or until death. Reported are clinical outcomes and treatment patterns in Mexico.

**Table 2.**
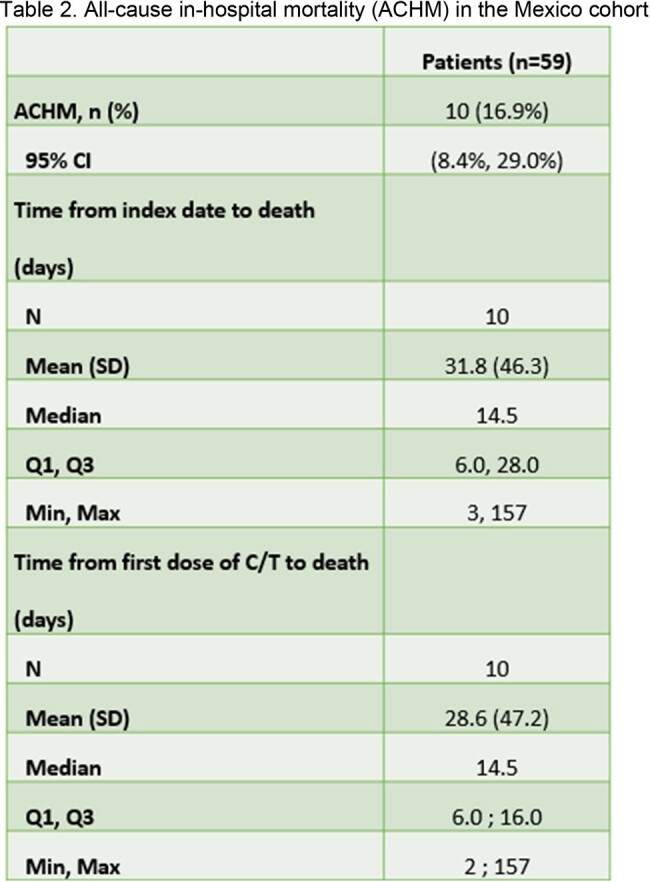
All-cause in-hospital mortality (ACHM) in the Mexico cohort

**Results:**

Mean age of patients (n=59) is 55.2 years, and 47.5% were female. Common comorbidities were diabetes mellitus (DM) uncomplicated (18.6%) and DM with end organ damage (3.4%), transplantation (16.9%), heart disease (16.9%), chronic kidney disease (11.9%), chronic pulmonary disease (10.2%), acute kidney injury (10.2%), and end-stage renal disease (3.4%). From positive cultures (n=32) were found 21 multi-drug resistant (MDR) *Pseudomonas aeruginosa (PsA)* (65.6%) and 4 MDR Enterbacterecea (12.5%). C/T was used to treat pneumonia (30/59, 50.8%), complicated intra-abdominal infection (9/59,15.3%), complicated urinary tract infection (8/59, 13.6%), bone and joint infection (8/59, 13.6%), and sepsis (8/59, 13.6%). Mean time from the index hospitalization admission to C/T initiation is 16.0 days. Mean time from the first microbiology (MB) sample for the index infection to C/T initiation is 1.9 days. C/T treatment duration was median 8.0 days (Q1:Q3; 5, 11). Doses included 54.2% 1.5g/Q8h; 39.0% 3g/Q8h; 5.1% 750mg/Q8h; 1.7% 1g/Q8h. C/T rank of initiations: first-line (49.2%), second-line (42.4%), third-line (6.8%), fourth-line (0), fifth-line (1.7%), sixth+line (0). Of all the cases, empiric therapy accounted for (37/59) 62.7%, definitive therapy in (17/59) 28.8%, and undetermined (5/59) (8.5%).

Clinical success rate for treating the index infection with C/T is 79.7% of patients (Table 1). All-Cause-Hospital-Mortality (ACHM) is 16.9%. Median time from index date to death is 12.0 days (Table 2). ICU admission is reported in 42.4% of cases, with a median ICU length of stay at 20.0 days (Table 3).

**Table 3.**
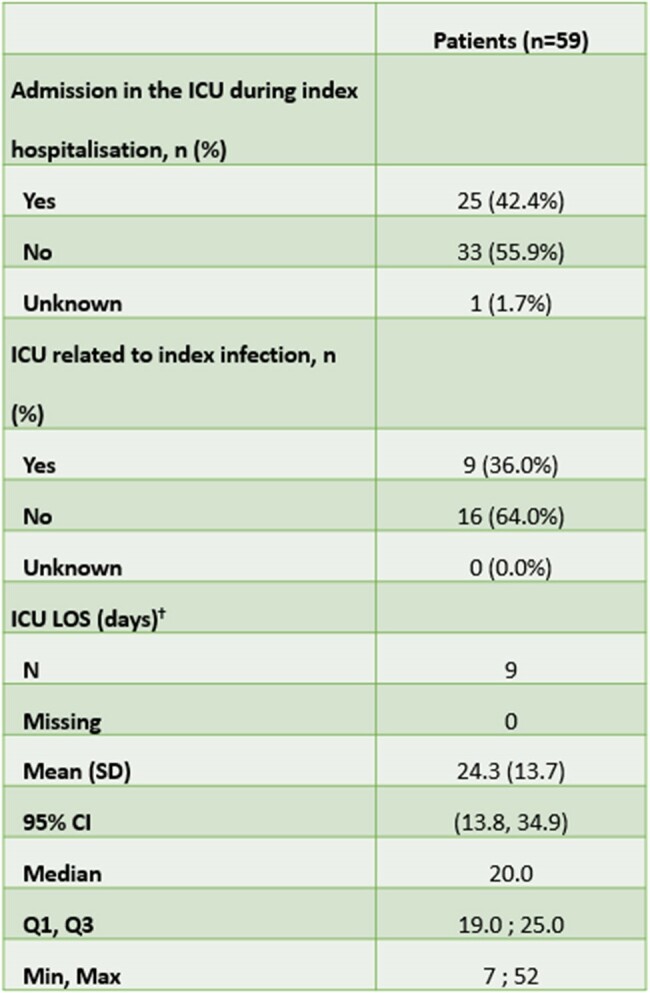
ICU admission and LOS during the index hospitalisation from the Mexico † ICU LOS if ICU admission was related to index infection. CI, confidence interval; ICU, intensive care unit; LOS, length of stay; SD, standard deviation.

**Conclusion:**

Results show real-world effectiveness of C/T in Mexico, more research is warranted.

**Disclosures:**

**Yanbing Zhou, PhD**, Merck: I am a full time Merck Employee and own stocks in the retirement plan provided by Merck.|Merck: Stocks/Bonds (Public Company) **Thales Polis, MD**, MSD: Stocks/Bonds (Public Company) **Emre Yucel, PhD**, Merck: I am a full time Merck Employee and own stocks in the retirement plan provided by Merck.|Merck: Stocks/Bonds (Public Company)

